# *TET1* Depletion Induces Aberrant CpG Methylation in Colorectal Cancer Cells

**DOI:** 10.1371/journal.pone.0168281

**Published:** 2016-12-15

**Authors:** Masahiro Kai, Takeshi Niinuma, Hiroshi Kitajima, Eiichiro Yamamoto, Taku Harada, Hironori Aoki, Reo Maruyama, Mutsumi Toyota, Yasushi Sasaki, Tamotsu Sugai, Takashi Tokino, Hiroshi Nakase, Hiromu Suzuki

**Affiliations:** 1 Department of Molecular Biology, Sapporo Medical University School of Medicine, Sapporo, Japan; 2 Department of Gastroenterology and Hepatology, Sapporo Medical University School of Medicine, Sapporo, Japan; 3 Medical Genome Science, Research Institute for Frontier Medicine, Sapporo Medical University School of Medicine, Sapporo, Japan; 4 Department of Molecular Diagnostic Pathology, Iwate Medical University, Morioka, Japan; University of Navarra, SPAIN

## Abstract

Aberrant DNA methylation is commonly observed in colorectal cancer (CRC), but the underlying mechanism is not fully understood. 5-hydroxymethylcytosine levels and *TET1* expression are both reduced in CRC, while epigenetic silencing of *TET1* is reportedly associated with the CpG island methylator phenotype. In the present study, we aimed to clarify the relationship between loss of *TET1* and aberrant DNA methylation in CRC. Stable *TET1* knockdown clones were established using Colo320DM cells, which express high levels of *TET1*, and HCT116 cells, which express *TET1* at a level similar to that in normal colonic tissue. Infinium HumanMethylation450 BeadChip assays revealed increased levels of 5-methylcytosine at more than 10,000 CpG sites in *TET1*-depleted Colo320DM cells. Changes in DNA methylation were observed at various positions within the genome, including promoters, gene bodies and intergenic regions, and the altered methylation affected expression of a subset of genes. By contrast, *TET1* knockdown did not significantly affect DNA methylation in HCT116 cells. However, *TET1* depletion was associated with attenuated effects of 5-aza-2’-deoxycytidine on gene expression profiles in both cell lines. These results suggest that loss of *TET1* may induce aberrant DNA methylation and may attenuate the effect of 5-aza-2’-deoxycytidine in CRC cells.

## Introduction

Cancers are thought to develop through accumulation of genetic and epigenetic alterations. A well-documented epigenetic alteration in human malignancies is aberrant DNA methylation. In mammals, DNA methylation is catalyzed by DNA methyltransferases (DNMTs), mainly at the C-5 position of cytosine (5-mC) in CpG dinucleotides. Cancer cells typically exhibit two patterns of abnormal DNA methylation: regional hypermethylation and global hypomethylation. Hypermethylation at gene promoter regions, especially CpG islands, is one of the major mechanisms by which tumor-related genes are inactivated in cancer. Moreover, a subset of cancers are characterized by concurrent hypermethylation at a number of CpG islands, which is termed the CpG island methylator phenotype (CIMP) [1]. The second pattern, global hypomethylation, is also commonly observed in malignancies, and can result in activation of oncogenes and retrotransposons, loss of imprinting and chromosomal instability.

Evidence emerging in recent years has shown that the ten-eleven translocation (TET) proteins play key roles in the mediation of active DNA demethylation. Members of the TET family (TET1-TET3) are oxoglutarate- and iron-dependent dioxygenases that catalyze the oxidation of 5-mC to generate 5-hydroxymethylcytosine (5-hmC) [2]. Further, sequential oxidation of 5hmC by TET generates 5-formylcytosine (5-fC) and 5-carboxylcytosine (5-caC), which can be removed by thymine DNA glycosylase. Deamination of 5-hmC by the deaminases AID and APOBEC followed by base-excision repair could also promote active demethylation of DNA.

The TET proteins and 5-hmC are critical to the regulation of pluripotency and differentiation potential in embryonic stem cells and induced pluripotent stem cells. Recent studies have shown that dysregulation of TET and 5-hmC levels could lead to carcinogenesis. Decreased TET expression and loss of 5-hmC are observed in various human malignancies, including melanoma and breast, lung, hepatic, gastric and esophageal cancers [3–7]. In addition, loss-of-function mutations in *TET2* and a resultant reduction in 5-hmC are observed in myeloid malignancies, including acute myeloid leukemia (AML), chronic myelomonocytic leukemia and myelodysplastic syndrome [8, 9]. There is also an important relationship between TET dysfunction and mutations in isocitrate dehydrogenase (IDH) family genes in several cancers. Somatic mutations in *IDH1* or *IDH2* result in the accumulation of an oncogenic metabolite, 2-hydroxyglutarate (2-HG), which can inhibit TET activity [10], and *IDH1/2* mutations are strongly associated with the hypermethylator phenotype in glioma and AML [11–13]. This suggests loss of TET function may increase CpG methylation through inhibition of active DNA demethylation.

A significant reduction of 5-hmC is reportedly observed in approximately 70% of colorectal cancer (CRC) cases [14]. Decreased *TET1* expression is found in approximately half of CRCs, and it is strongly associated with loss of 5-hmC [14]. TET1-catalyzed 5-hmC formation regulates gene expression during the differentiation of colonocytes, and altered 5-hmC levels may contribute to tumorigenesis [15]. TET1 also exhibits tumor suppressor function through regulation of the Wnt signaling pathway in colon cancer cells [16]. Although *IDH1/2* mutations have not been reported in CRC, whole exome sequencing analysis identified mutations in all three TET family genes in CRC [17]. Furthermore, we and others recently reported that *TET1* is silenced in association with promoter CpG island hypermethylation in a subset of CRC cell lines [18]. *TET1* methylation is preferentially observed in CIMP-positive primary CRCs as well as in CIMP-positive colorectal adenomas [18]. Methylation of TET1 CpG island in primary CRC is also reported by another group recently [19]. These observations suggest that loss of *TET1* function may induce aberrant DNA methylation during the development of CRC, although a direct link between loss of *TET1* and increased CpG methylation has not yet been confirmed in CRC cells. To clarify this issue, we assessed the effect of stable *TET1* knockdown on genome-wide DNA methylation and gene expression profiles in CRC cell lines. We observed that depletion of *TET1* leads to increased methylation at a number of CpG sites in a CRC cell line and may attenuate the effect of DNMT inhibition on gene expression profiles in CRC cells.

## Materials & Methods

### Cell lines

The Colo320DM, HCT116, CaCO2, DLD1, HT29, LoVo, RKO, SW48 and SW480 CRC cell lines were described previously [20], and the T84, WiDr and SW620 lines were purchased from the American Type Culture Collection (ATCC). Colo320DM and HCT116 cells were respectively maintained in DMEM or McCoy’s 5A medium supplemented with 10% feral bovine serum. For DNMT inhibition, cells were treated with 1 μM 5-aza-2’-deoxycytidine (5-aza-dC; Sigma-Aldrich, St Louis, MO, USA) for 72 h, replacing the drug and medium every 24 h. Genomic DNA was extracted using the standard phenol-chloroform method. Total RNA was extracted using TRI Reagent (COSMO BIO, Tokyo, Japan).

### Quantitative reverse-transcription PCR

Single-stranded cDNA was prepared using SuperScript III reverse transcriptase (Thermo Fisher Scientific, Waltham, MA, USA), after which the integrity of the cDNA was confirmed by amplifying glyceraldehydes-3-phosphate dehydrogenase (*GAPDH*). Quantitative reverse-transcription PCR (RT-qPCR) was carried out using TaqMan Gene Expression Assays (*TET1*, Hs00286756_m1; *GAPDH*, Hs02758991_g1; Thermo Fisher Scientific) and a 7500 Fast Real-Time PCR System (Thermo Fisher Scientific). SDS ver. 1.4 (Thermo Fisher Scientific) was used for comparative delta Ct analysis.

### Stable knockdown of *TET1* in CRC cells

RNAi-induced *TET1* knockdown was accomplished using a BLOCK-iT Pol II miR RNAi Expression Vector kit (Thermo Fisher Scientific). Two sets of oligonucleotides targeting TET1 were purchased from Invitrogen and ligated into a pcDNA6.2-GW/EmGFP-miR vector (Thermo Fisher Scientific). Cells (3 × 10^5^ cells in 6-well plates) were transfected with each TET1 knockdown vector (miTET1-1 or miTET1-2) or a pcDNA6.2-GW/EmGFP-miR-neg control plasmid (Thermo Fisher Scientific), after which cells were selected for 2 weeks with 1.0 mg/ml (Colo320DM) or 0.6 mg/ml (HCT116) G418. GFP-positive colonies were isolated, and knockdown efficiencies were analyzed using RT-qPCR. *TET1* knockdown clones and control clones were then cultured for an additional 2 to 3 weeks to obtain a sufficient number of cells for experimentation.

### Cell viability assay

Cell proliferation was assessed by measuring the uptake of a water-soluble tetrazolium salt in WST-8 assays, as described previously [20]. Briefly, transfected cells were seeded onto 96-well plates to a density of 5 × 10^3^ cells per well. After incubation for 24 h, 48 h or 72 h, WST-8 assays were carried out using a Cell Counting kit-8 (Dojindo, Tokyo, Japan) according to the manufacturer's instructions.

### Dot blot analysis

Genomic DNA samples (500 ng) were spotted onto Hybond N+ nylon membranes (GE Healthcare, Tokyo, Japan). The blotted membrane was dampened with TE and then applied to a UV-crosslink procedure (1200 J/cm^2^, 30 sec) using Stratalinker 2400 (Agilent Technologies, Santa Clara, CA, USA). The membrane was then blocked with 100% BlockAce (DS Pharma Biomedical, Osaka, Japan) for 30 min at room temperature and incubated with a mouse anti-5-methylcytosine (5-mC) monoclonal antibody (1:1,000 dilution, Catalog No 39649, Active Motif, Carlsbad, CA, USA), with a rabbit anti-5-hydroxymethylcytosine (5-hmC) polyclonal antibody (1:10,000 dilution, Catalog No 39769, Active Motif), or with normal mouse IgG (1 μg/mL, Santa Cruz Biotechnology, Santa Cruz, CA, USA) overnight at 4°C. Thereafter, the membrane was incubated with HRP-conjugated anti-mouse IgG (1:10,000, The Jackson Laboratory, Bar Harbor, ME, USA) for 1 h at room temperature. The chemiluminescent signals were detected using ECL kits (GE Healthcare, Little Chalfont, UK) and an ImageQuant LAS4000 mini detection system (GE Healthcare).

### Bisulfite pyrosequencing and bisulfite sequencing analysis

Genomic DNA (1 μg) was modified with sodium bisulfite using an EpiTect Bisulfite Kit (Qiagen, Hilden, Germany), after which bisulfite pyrosequencing and bisulfite sequencing were carried out as described [20]. For bisulfite pyrosequencing, the biotinylated PCR product was purified, made single-stranded and used as a template in a pyrosequencing reaction run according to the manufacturer’s instructions. The pyrosequencing reaction was carried out using a PSQ96 system with a PyroGold reagent Kit (Qiagen), and the results were analyzed using Q-CpG software (Qiagen). For bisulfite sequencing, amplified PCR products were cloned into pCR2.1-TOPO vector (Life Technologies), and 10 clones from each sample were sequenced using an ABI3130x automated sequencer (Life Technologies). Primer sequences and PCR product sizes are listed in S1 Table.

### Infinium assay

Genome-wide DNA methylation was analyzed using an Infinium HumanMethylation450 BeadChip (Illumina, San Diego, CA, USA) as described elsewhere. The data were then assembled using GenomeStudio methylation software (Illumina). Array data ware imported into Gene Spring GX ver. 13 (Agilent Technologies), after which statistical analysis and gene ontology (GO) analysis were carried out. Probes differentially methylated between control and knockdown cells were selected using t tests with Benjamini-Hochberg correction (P < 0.05). Results from selected genes were visualized using the University of California Santa Cruz (UCSC) genome browser. The Gene Expression Omnibus accession number for the Infinium assay data is GSE84400.

### Gene expression microarray

Total RNA (100 ng) was amplified and labeled using a Low Input Quick Amp Labeling kit one-color (Agilent Technologies), after which the synthesized cRNA was hybridized to a SurePrint G3 Human GE 8 × 60K Ver 2.0 microarray (G4851B; Agilent technologies). Data analysis was carried out using GeneSpring GX ver. 13 (Agilent technologies). Probes differentially expressed between control and knockdown cells were selected by performing t tests with the Benjamini-Hochberg correction (P < 0.05). The Gene Expression Omnibus accession number for the microarray data is GSE84400.

### Statistical analysis

To compare differences in continuous variables between groups, one-way ANOVA with post hoc tests was performed. Values of *P* < 0.05 (two-sided) were considered statistically significant. Statistical analyses were carried out using GraphPad Prism 5 (GraphPad software, La Jolla, CA, USA).

## Results

### Establishment of stable *TET1* knockdown CRC cells

**Fig 1:**
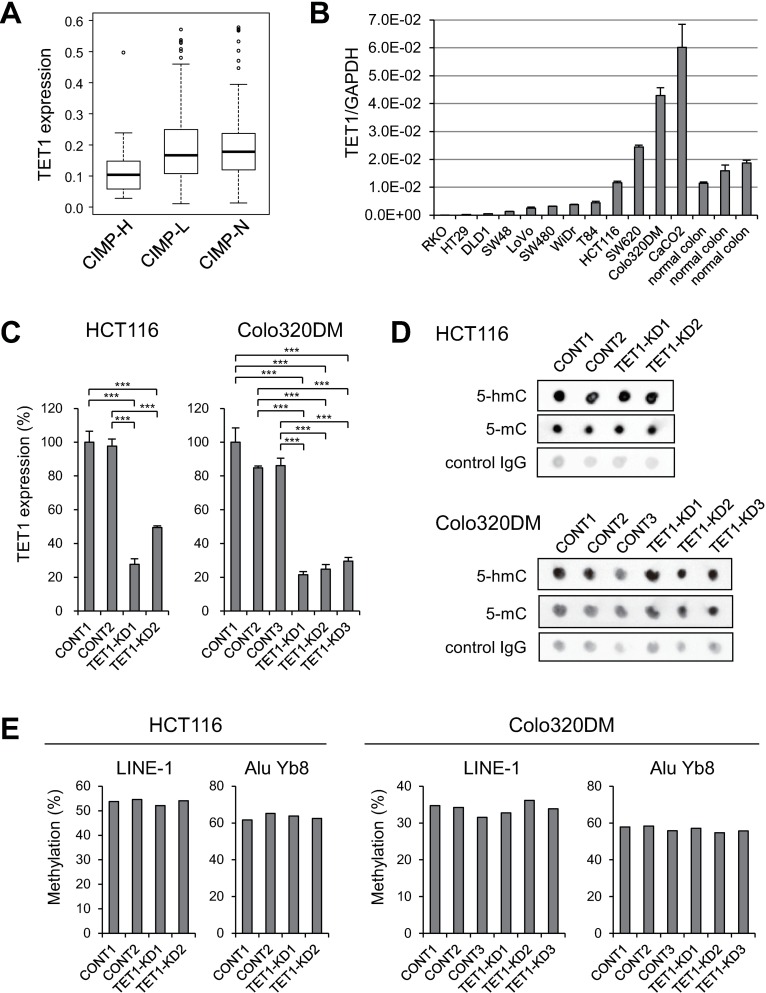
Establishment of stable *TET1* knockdown CRC cells. (A) Summaries of *TET1* expression in CIMP-high (CIMP-H), CIMP-low (CIMP-L) and CIMP-negative (CIMP-N) CRCs in TCGA datasets. (B) Results of RT-qPCR analyses of *TET1* in the indicated CRC cell lines and normal colonic tissues. (C) RT-qPCR analysis of *TET1* in control clones and stable *TET1* knockdown clones of the indicated CRC cells. ***P < 0.001 (one-way ANOVA with post hoc tests). (D) Dot blot analysis of 5-hydroxymethylcytosine (5-hmC) and 5-methylcytosine (5-mC) in the indicated clones. Results using a control IgG are shown below as loading controls. (E) Bisulfite pyrosequencing analysis of repetitive elements in the indicated clones.

To assess the expression levels of *TET1* in CRCs, we first used RNA-seq data obtained from primary CRC tissues in The Cancer Genome Atlas (TCGA) network study. We found that CRCs in the CIMP-high category showed significantly lower levels of *TET1* expression than CIMP-low or CIMP-negative tumors (Fig 1A), which is consistent with previous observations [18]. We next carried out RT-qPCR with *TET1* in a series of CRC cell lines, and found that the majority of CRC lines (8 of 12) expressed lower levels of *TET1* than normal colonic tissues, whereas two lines (Colo320DM and CaCO2) showed upregulated *TET1* expression (Fig 1B). To explore the effect of *TET1* downregulation in CRC cells, we selected for knockdown experiments HCT116 cells, in which *TET1* is expressed at a level similar to that in normal colon, and Colo320DM cells, in which *TET1* is expressed at a higher level than in normal colon. These cells were transfected with a plasmid encoding an artificial microRNA (miRNA) targeting *TET1* or a negative control, after which the transfectants were selected for 5 to 6 weeks. Using two different artificial miRNAs targeting *TET1* (miTET1-1 and miTET1-2), we established two stable *TET1* knockdown clones of HCT116 cells (TET1-KD1 and TET1-KD2) (Fig 1C). We also isolated two native control clones (CONT1 and CONT2). In TET1-KD1 cells, TET1 mRNA levels were reduced by about 70% as compared to control, while in TET1-KD2 cells they were reduced by approximately 50%. With Colo320DM cells, we also established three independent knockdown clones using the two miRNAs (miTET1-1 for TET1 KD1 and KD2; miTET1-2 for KD3) and three control clones (CONT1, CONT2 and CONT3) (Fig 1C). Cell viability assays revealed that TET1 depletion had no significant effect on cell proliferation in either HCT116 or Colo320DM cells (Fig A in S1 File).

Using dot blot analysis, we evaluated global 5-hmC and 5-mC levels in the control and *TET1* knockdown clones (Fig 1D). Unexpectedly, we found that depletion of *TET1* did not induce significant changes in 5-hmC levels in either HCT116 or Colo320DM cells. Similarly, *TET1* depletion had no effect on 5-mC levels in HCT116 cells. By contrast, it appeared 5-mC levels were higher in the Colo320DM *TET1* knockdown clones than in control clones (Fig 1D). Previous studies showed that methylation of repetitive elements could be a surrogate marker of global 5-mC levels [21]. We therefore used quantitative bisulfite pyrosequencing to assess the methylation of LINE-1 and Alu Yb8, but detected no significant differences between control and knockdown clones with either HCT116 or Colo320DM cells (Fig 1E).

### Increased CpG methylation in *TET1* depleted Colo320DM cells

**Fig 2:**
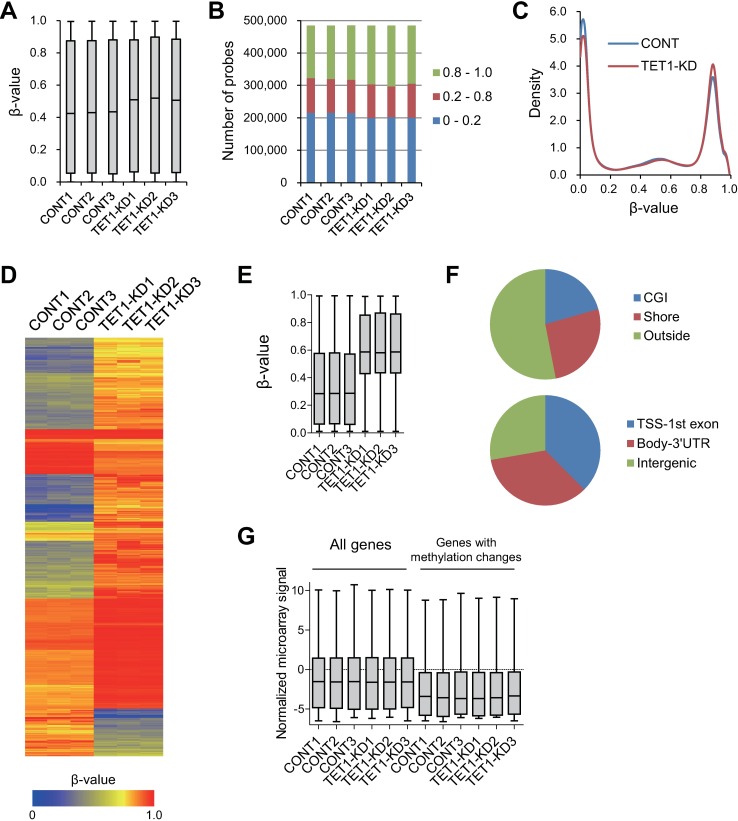
Changes in DNA methylation induced by *TET1* depletion in Colo320DM cells. (A) Box plots showing the β-values of all probe sets from an Infinium HumanMethylation450 BeadChip for Colo320DM control or *TET1* knockdown clones. (B) Numbers of probe sets on an Infinium BeadChip with the indicated β-values in control and *TET1* knockdown clones. (C) Density plots showing BeadChip results for control and *TET1* knockdown clones. Shown are averages of three independent clones each. (D) Heatmap of the BeadChip probe sets differentially methylated between control and *TET1* knockdown clones. (E) Box plots showing the β-values of differentially methylated probes in the indicated clones. *P* < 0.001 (one-way ANOVA). (F) Genomic locations of the differentially methylated probes. (G) Box plots showing the expression levels of all genes or genes associated with differentially methylated probes (selected genes) in the indicated clones. *P* < 0.001 (one-way ANOVA).

To obtain more detailed insight into genome-wide 5-mC levels, we carried out Infinium HumanMethylation450 BeadChip assays. Consistent with the dot blots and bisulfite pyrosequencing, there were no apparent differences in the methylation status between HCT116 control and *TET1* knockdown clones (Fig B in S1 File). However, overall β-values were increased in the Colo320DM *TET1* knockdown clones, which is consistent with the 5-mC levels determined in the dot blot analysis (Fig 2A–2C). Median β-values for all probes in the control clones ranged from 0.42 to 0.44, while those for the knockdown clones ranged from 0.51 to 0.52 (Fig 2A). We observed a substantial increase (approximately 15000 probes) in the number of methylated probes (β-value ≥ 0.8) and a similar sized decrease in the number of unmethylated probes (β-value < 0.2) in the *TET1* knockdown clones (Fig 2B and 2C). We therefore extracted probe sets in which the methylation status recurrently differed between the control and knockdown clones (*P* < 0.05). Among the 3920 extracted probe sets, the majority showed higher methylation levels in the knockdown clones than in the control clones (Fig 2D and 2E). In approximately half the probes, the methylation changes were located in the CpG islands (CGIs) or CGI shores, while the remaining probes were outside of the CpG island regions (Fig 2F). In addition, probes with methylation changes were widely distributed in various genomic positions, including both genic and intergenic regions (Fig 2F). Of the 3920 probes with methylation changes, 2698 were located in genic regions (corresponding to 2019 unique genes). Gene ontology analysis revealed that genes associated with “cell periphery”, “plasma membrane” and “system development” were significantly enriched among these genes (S2 Table). However, microarray analysis carried out to assess the gene expression profiles in these cells revealed no significant differences in the expression levels of the majority of these genes between the control and knockdown clones (Fig 2G). However, it is notable that the expression levels of the genes with methylation changes were strikingly lower than the overall expression level of all protein coding genes (Fig 2G).

### Altering DNA methylation affects gene expression in Colo320DM cells

**Fig 3:**
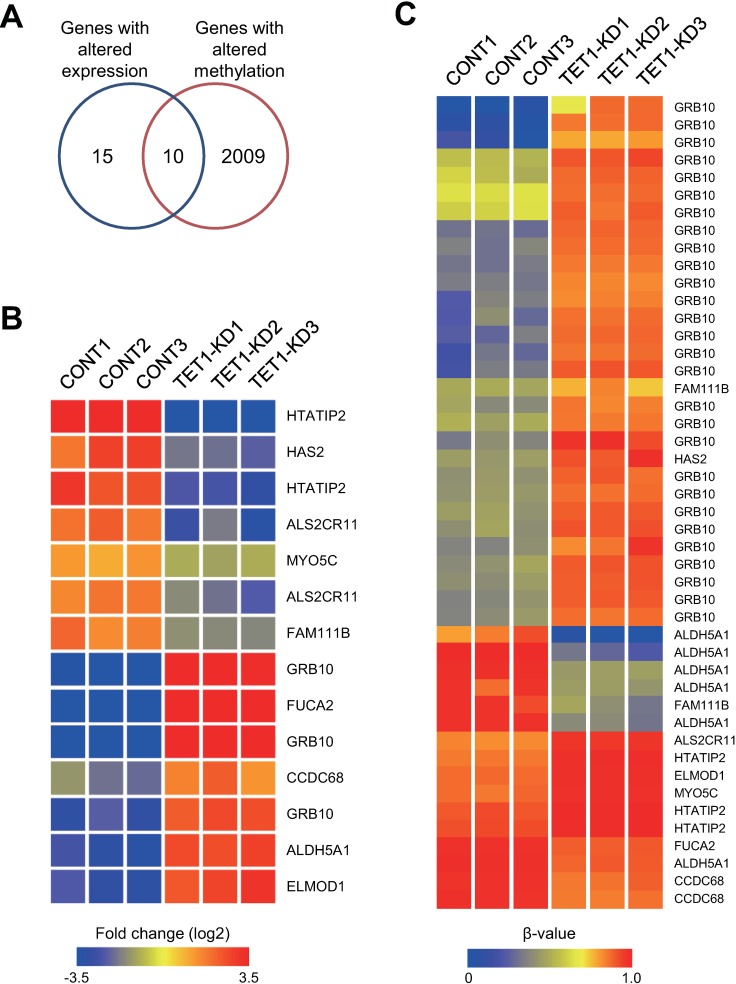
Association between DNA methylation changes and gene expression in *TET1* knockdown Colo320DM cells. (A) Venn diagram for genes with altered DNA methylation and genes with altered expression in *TET1* knockdown cells. (B) Heatmap of the expression of the 10 selected genes in (A). (C) Heatmap of the Infinium BeadChip probes for the 10 selected genes.

To examine the effects of *TET1* depletion in more detail, we further analyzed the Infinium BeadChip data and microarray results obtained with the control and *TET1* knockdown Colo320DM cells. We found that expression of only a small number of genes significantly differed between the control and knockdown clones (29 probes corresponding to 25 unique genes, *P* < 0.05), and a majority of those were downregulated in the *TET1* knockdown clones (Fig C in S1 File). Of that group, 10 genes overlapped those with altered DNA methylation (Fig 3, Table 1).

**Table 1:** Genes with altered methylation and expression in TET1 knockdown Colo320DM cells.

Gene name	Locus	Methylation	Location of altered methylation	CpG island	Expression
ALS2CR11	2q33.1	Up	TSS-1st Exon	Island	Down
ALDH5A1	6p22.3	Down	TSS-1st Exon	Island	Up
		Down	Body	Island	
FUCA2	6q24.2	Down	TSS-1st Exon	Island	Up
GRB10	7p12.1	Up	TSS-1st Exon	Shore	Up
		Up	Body		
		Up	3'UTR		
HAS2	8q24.13	Up	TSS-1st Exon	Shore	Down
HTATIP2	11p15.1	Up	TSS-1st Exon	Island	Down
FAM111B	11q12.1	Up	TSS-1st Exon	Shore	Down
		Down	Body		
ELMOD1	11q22.3	Up	TSS-1st Exon		Up
		Up	3'UTR		
MYO5C	15q21.2	Up	TSS-1st Exon	Shore	Down
CCDC68	18q21.2	Down	TSS-1st Exon	Island	Up

**Fig 4:**
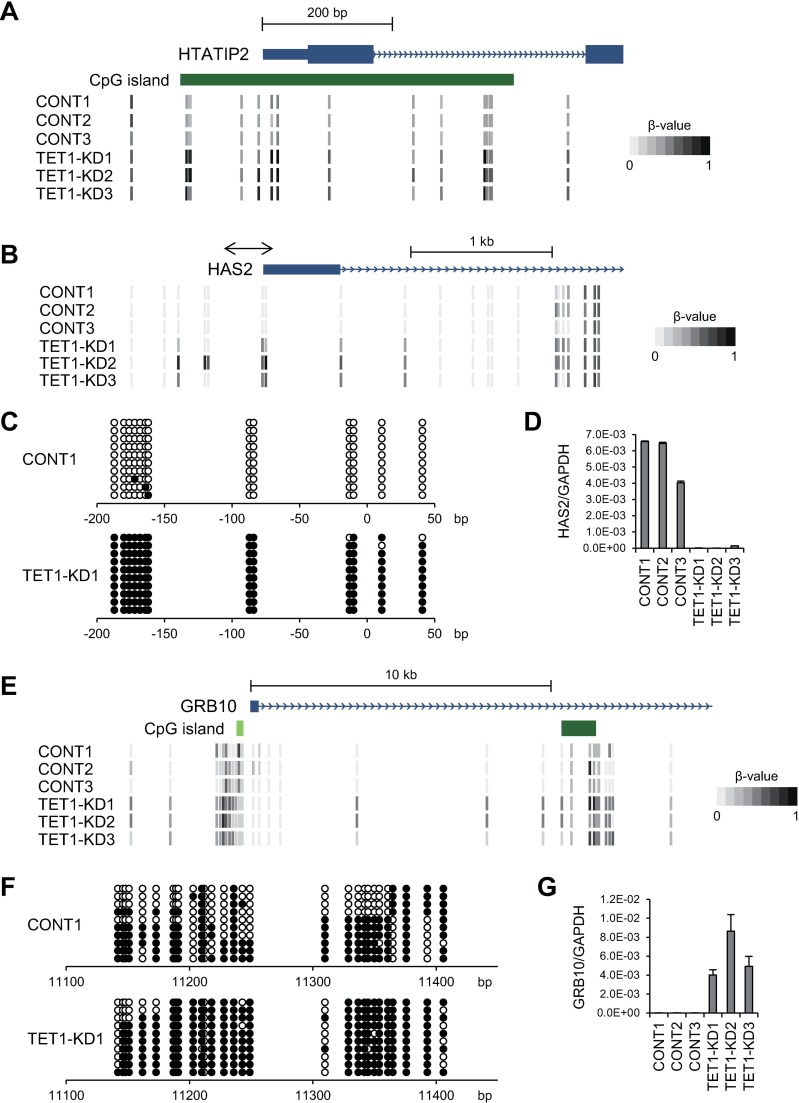
Analysis of DNA methylation and expression of representative genes in Colo320DM cells. (A) Diagram of the Infinium BeadChip results around the transcription start site (TSS) of *HTATIP2* in control and *TET1* knockdown Colo320DM cells. (B) Infinium BeadChip results around the TSS of *HAS2*. The region analyzed with bisulfite sequencing is indicated on the top. (C) Bisulfite sequencing analysis of *HAS2*. Open and filled circles represent unmethylated and methylated CpG sites, respectively. (D) RT-qPCR analysis of *HAS2*. (E) Infinium BeadChip results around the first exon and intron of *GRB10*. (F) Bisulfite sequencing analysis of the intragenic CpG island of *GRB10*. (G) RT-qPCR analysis of *GRB10*.

There was a positive relationship between upregulated methylation in the 5’ regions (transcription start sites and 1st exons) and downregulated expression of five genes (*HAS2*, *HTATIP2*, *MYOC5C*, *ALS2CR1* and *FAM111B*) in the *TET1* knockdown clones (Fig 3B and 3C). For instance, the promoter CpG island of *HTATIP2* was moderately methylated in the control clones, whereas the entire CpG island is nearly completely methylated in the *TET1* knockdown clones (Fig 4A). The *HAS2* promoter is not associated with a CpG island, but levels of DNA methylation around the transcription start site were strikingly elevated in *TET1* knockdown clones (Fig 4B). The methylation status of *HAS2* was further validated by bisulfite sequencing (Fig 4C, Fig. D in S1 File), and RT-qPCR confirmed its downregulated expression in the knockdown clones (Fig 4D). Conversely, the promoter CpG islands of three genes (*FUCA2*, *CCDC68* and *ALDH5A1*) showed decreased methylation and upregulated expression in *TET1* knockdown clones (Fig 3B and 3C).

Increased methylation within the gene body regions of two genes (*GRB10* and *ELMOD1*) was associated with upregulated expression in *TET1* knockdown clones (Fig 3). Notably, in GRB10, methylation levels were significantly increased over the entire gene body region, including the intragenic CpG island (Fig 4E, Fig E in S1 File). *GRB10* is an imprinting gene, and the intragenic CpG island is known as a differentially methylated region (DMR), which is normally methylated only in the maternal allele [22]. Bisulfite sequencing confirmed that the CpG island was almost completely methylated in the *TET1* knockdown cells, while the region was methylated at about half that level in the control cells (Fig 4F, Fig D in S1 File). Elevated *GRB10* expression in *TET1* knockdown clones was confirmed by RT-qPCR (Fig 4G). In addition, by performing statistical analyses with less stringent criteria, we detected additional genes with altered methylation and expression in *TET1* knockdown cells (Fig F in S1 File, S3 Table). For instance, *PDGFRB* was downregulated in association with promoter hypermethylation, and *GRIP1* was upregulated with gene body methylation in the *TET1* knockdown clones (Fig G in S1 File).

### *TET1* depletion attenuates the response to a DNMT inhibitor

**Fig 5:**
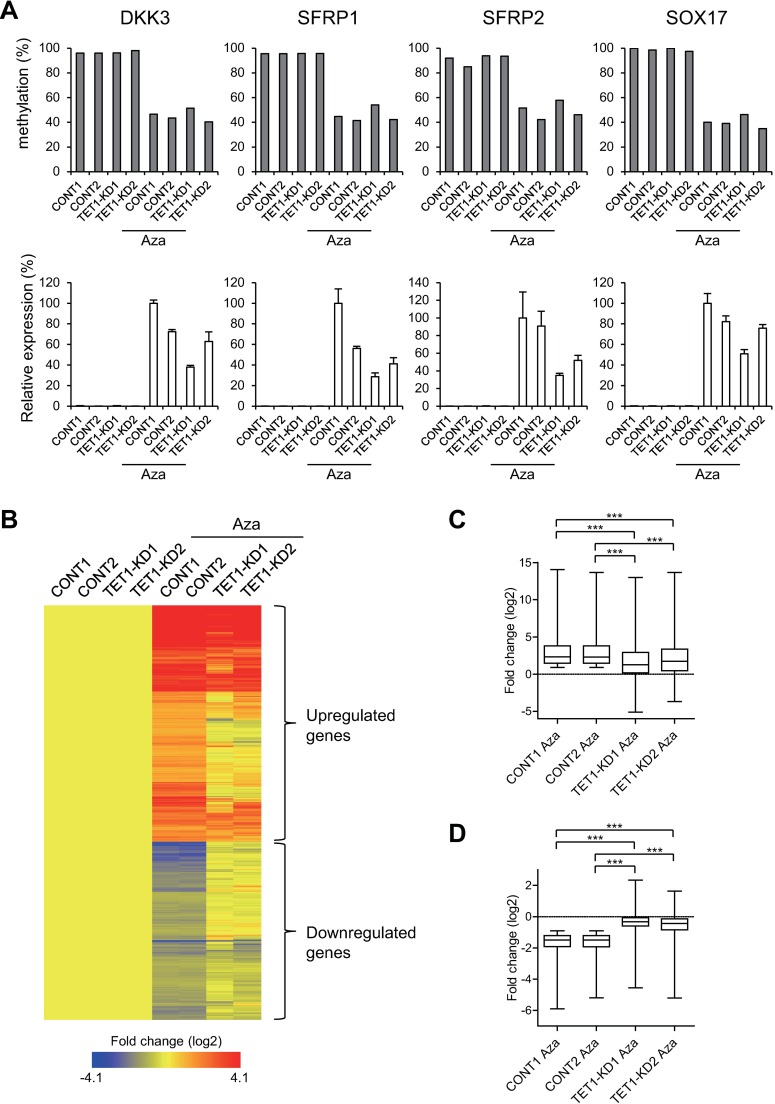
Diminished effects of 5-aza-dC (Aza) in *TET1*-depleted HCT116 cells. (A) Analysis of DNA methylation (upper panel) and expression of representative tumor suppressor genes (lower panel) in control and *TET1* knockdown HCT116 cells treated with or without Aza. Methylation levels at 2 CpG sites in *DKK3*, 3 CpG sites in *SFRP1*, 4 CpG sites in *SFRP2* or 3 CpG sites in *SOX17* were analyzed using bisulfite pyrosequencing, and averages of respective genes are shown (upper panel). RT-qPCR results are shown relative to the expression levels in the Aza-treated CONT1 clone, which was assigned a value of 100 (lower panel). (B) Heatmap showing the expression of genes affected by Aza in HCT116 cells. Genes whose expression was altered after Aza in control clones were selected, and hierarchical clustering was performed. (C) Box plot showing the expression levels of genes upregulated by Aza. ***P < 0.001 (one-way ANOVA with post hoc tests). (D) Box plot showing the expression levels of genes downregulated by Aza. ***P < 0.001 (one-way ANOVA with post hoc tests).

As described above, *TET1* knockdown had only a limited effect on 5-mC in HCT116 cells. Because Infinium BeadChip assays indicated that global DNA methylation levels are significantly higher in HCT116 than Colo320DM cells (median β-values of all probes were 0.86 to 0.87 in HCT116 vs. 0.42 to 0.44 in Colo320DM), *TET1* depletion may be unable to induce further DNA methylation in HCT116 cells. A recent study showed that in hepatocellular carcinoma (HCC) cells, active DNA demethylation using a DNMT inhibitor is dependent on *TET2* [23]. We therefore tested the effect of *TET1* depletion on pharmacological DNA demethylation and gene re-expression in HCT116 cells by treating control and *TET1* knockdown clones with or without 5-aza-dC, and analyzing the expression and DNA methylation of four representative tumor suppressor genes: *DKK3*, *SFRP1*, *SFRP2* and *SOX17*. These genes are normally silenced in HCT116 cells in association with promoter CpG island methylation [24–26]. The degrees to which 5-aza-dC treatment induced re-expression of the four genes were lowest in the TET1-KD1 clone, where the *TET1* depletion was greatest (Figs 1C and 5A). Quantitative DNA methylation analysis using bisulfite pyrosequencing revealed that the four genes were almost fully methylated in both control and *TET1* knockdown clones, and the highest levels of methylation after 5-aza-dC treatment were in the TET1-KD1 clone, though the differences were modest (Fig 5A). These results suggest that *TET1* depletion in CRC cells may be associated with attenuated responses to 5-aza-dC.

To confirm the relationship between *TET1* and the drug effect, we carried out a gene expression microarray analysis using the HCT116 control and *TET1* knockdown clones treated with or without 5-aza-dC. We found that 5-aza-dC induced significant changes in the gene expression profiles of the control clones. Statistical analysis revealed that 3037 probe sets (corresponding to 2585 unique genes) were upregulated, while 2291 probe sets (1923 unique genes) were downregulated by the treatment (*P* < 0.05). Hierarchical clustering analysis of the affected genes clearly demonstrated that the magnitudes of the changes in gene expression were markedly smaller in the *TET1* knockdown clones than controls (Fig 5B). Box plots of the expression levels of upregulated (Fig 5C) and downregulated genes (Fig 5D) suggest the effect of 5-aza-dC on gene expression was diminished in the *TET1* knockdown clones. A microarray analysis of Colo320DM cells treated with 5-aza-dC yielded similar results (Fig H in S1 File).

## Discussion

We previously showed that the *TET1* gene is preferentially methylated in CIMP-positive and *BRAF* mutant CRCs and adenomas, which suggests *TET1* is one of the genes affected by CIMP-related hypermethylation [18]. From those results, however, we could not determine whether *TET1* methylation is merely a consequence of CIMP or whether loss of *TET1* contributed to aberrant DNA methylation. To test the effect of long-term *TET1* depletion, we selected two CRC cell lines in which *TET1* is unmethylated and expressed. HCT116 cells are a MSI-positive cell line in which the mismatch repair genes *MLH1* and *MSH3* are mutated [27]. Aberrant DNA methylation is heavy in HCT116 cells (possibly CIMP-positive) [28], but they retain wild-type *BRAF* and show *KRAS* mutation instead. Colo320DM is a MSS cell line in which *TP53* is mutated and both *KRAS* and *BRAF* are wild-type. What causes *TET1* to be overexpressed in Colo320DM cells is unclear, but a recent study in human bronchial epithelial cells showed that KRAS activation suppresses *TET1* expression, which in turn triggers DNA hypermethylation and cellular transformation [29]. That finding appears consistent with our observation that the two CRC cell lines with wild-type *KRAS* had the highest *TET1* expression levels (CaCO2 and Colo320DM), although the biological relevance of the TET1 upregulation in these cells remains to be clarified.

We obtained several unexpected results from the stable *TET1* knockdown CRC cells. For instance, we detected no significant changes in 5-hmC levels after *TET1* depletion. In addition, depletion of *TET1* had no effect on CRC cell proliferation, though in one recent study *TET1* exerted tumor suppressive effects in CRC cells [16]. One possible explanation for this apparent discrepancy is that, during the long-term culture needed to establish stable knockdown clones, the reduction in 5-hmC was compensated via another mechanism, though we observed no upregulation of other *TET* family members in the knockdown cells (data not shown). Alternatively, the sensitivity of our dot blot analysis may not have been sufficient to detect changes in 5-hmC levels at the respective loci. These results may thus indicate a limitation of our study, in which we could not fully recapitulate the *TET1* downregulation in primary CRC.

Infinium BeadChip analysis revealed that levels of 5-mC were increased at as many as 15000 CpG sites in *TET1*-depleted Colo320DM cells (approximately 3.3% of the CpG sites covered by HumanMethylation450 BeadChip). We also noted that the genes exhibiting methylation changes were strongly associated with “development” and were expressed at lower levels in CRC cells. This may be consistent with the recent finding that TET proteins are key regulators of stem cell differentiation [30]. Our observations also appear to correspond with the earlier report that methylation-prone genes are downregulated in cancer and that they function in developmental processes [31]. Similarly, in gastric mucosa, genes susceptible to aberrant methylation induced by *Helicobacter pylori* infection are expressed at lower levels than the methylation-resistant genes [32].

Despite the apparent effects on DNA methylation, *TET1* depletion exerted only moderate effects on gene expression. Methylation changes induced by *TET1* depletion were located at various positions within the genome in Colo320DM cells. In many of the affected genes, the numbers of CpG sites with methylation changes were relatively small and perhaps insufficient to affect gene expression. Nevertheless, both methylation and expression of a subset of important tumor-related genes were significantly altered in *TET1*-depleted CRC cells. *HTATIP2* (also known as *TIP30* or *CC3*) was first identified as a metastasis suppressor in breast cancer [33] and was often found to be downregulated in various cancers. This downregulation of *HTATIP2* is reportedly associated with promoter CpG island methylation in HCC, CRC, esophageal squamous cell carcinoma and glioma [34–38]. *HAS2*, which encodes hyaluronan synthase 2, is overexpressed in multiple tumor types, including breast and prostate cancer [39, 40], and HAS2 reportedly promotes cancer cell invasion and metastasis [41, 42]. It was recently shown that treating pancreatic ductal carcinoma cells with 5-aza-dC leads to increased expression of *HAS2*, suggesting an epigenetic mechanism is involved in regulating *HAS2* in cancer cells [43].

In contrast to the genes mentioned above, we found that increased methylation in the gene body of *GRB10* is associated with its upregulated expression in *TET1* knockdown Colo320DM cells. GRB10 was first identified as an epidermal growth factor receptor (EGFR) binding protein [44], and subsequent studies revealed that GRB10 also interacts with multiple receptor tyrosine kinases and intracellular kinases. Expression of *GRB10* is upregulated in melanoma, cervical cancer and AML [45–47]. GRB10 interacts with oncogenic Bcr-Abl in chronic myelogenous leukemia [48], and with active Raf-1 to promote cell survival [49], and it also binds with FLT3 to enhance AML cell proliferation [47], which is suggestive of oncogenicity. By contrast, GRB10 also acts as a tumor suppressor by negatively regulating phosphatidylinositol 3-kinase and insulin signaling [50], and is silenced in association with promoter methylation in renal cancer [51]. Recent studies have shown that elevated methylation in the gene body region is positively associated with gene expression [52–54]. Thus, gene body methylation may be a mechanism by which to upregulate *GRB10* in cancer cells, although the function of *GRB10* in CRC remains to be clarified.

Although *TET1* knockdown had only limited effects on DNA methylation in HCT116 cells, it appears responses to 5-aza-dC may be attenuated by *TET1* depletion in CRC cells. The magnitude of DNA demethylation and re-expression of epigenetically silenced tumor suppressor genes induced by 5-aza-dC were lower in *TET1*-depleted than control HCT116 cells. Moreover, the effects of 5-aza-dC on gene expression profiles were significantly diminished by *TET1* depletion in both HCT116 and Colo320DM cells. These results suggest that the pharmacological effect of 5-aza-dC may be, at least in part, dependent of TET1. While we were preparing this manuscript, another group reported that TET2 is required for DNA demethylation by 5-azacytidine in HCC cells [23]. They showed that 5-azacytidine treatment significantly increased expression of *TET2* and *TET3* in HCC cells, and *TET2* knockdown disrupted the generation of 5-hmC by 5-azacytidine, suggesting that 5-azacytidine could trigger TET2-dependent active demethylation. It is thus possible that TET1 plays a similar role in CRC cells treated with 5-aza-dC.

Another recent study showed that TET1 acts as a tumor suppressor by regulating the Wnt signaling pathway in CRC [16]. In CRC cells, re-expressed TET1 targets the *DKK* and *SFRP* genes, which encode upstream inhibitors of Wnt signaling. TET1 upregulates their expression by reducing 5-mC and increasing 5-hmC at their promoter regions [16]. Our results indicate that TET1 may participate in the 5-aza-dC-induced restoration of *DKK* and *SFRP* genes in CRC cells.

In summary, we have shown that depleting *TET1* may induce DNA methylation in CRC cells, suggesting downregulation of *TET1* may facilitate the accumulation of aberrant DNA methylation during the development of CRC. Our results also indicate that TET1 may contribute to the pharmacological effect of a DNMT inhibitor, suggesting that restoration of TET1 function would be a useful strategy for the treatment of CRC.

## Supporting Information

S1 File Fig A in S1 File. Cell viability assay results from control and *TET1* knockdown clones of the indicated CRC cell lines. Fig B in S1 File. Summary of Infinium HumanMethylation450 BeadChip assays with control and *TET1* knockdown HCT116 cells. Fig C in S1 File. Heatmap showing the gene expression microarray results from selected 25 genes that were differentially expressed between control and *TET1* knockdown clones of Colo320DM cells. Fig D in S1 File. Sequences of the regions analyzed with bisulfite sequencing and shown in Fig 4C and 4F. Fig E in S1 File. Diagram of the HM450 BeadChip results for the entire *GRB10* gene region in control and *TET1* knockdown clones of Colo320DM cells. Fig F in S1 File. Association between DNA methylation and gene expression in *TET1* knockdown Colo320DM cells. Fig G in S1 File. Analysis of DNA methylation and expression of selected genes in control and *TET1* knockdown Colo320DM cells. Fig H in S1 File. Attenuated effects of 5-aza-dC (Aza) on gene expression profiles in *TET1*-depleted Colo320DM cells.
(DOC)

S1 Table Sequences of the primers used in this study. (XLS)

S2 Table Gene ontology analysis of genes with methylation changes in *TET1* knockdown Colo320DM cells. (XLS)

S3 Table Genes with altered methylation and expression in *TET1* knockdown Colo320DM cells. (XLS)
