# Supplementary material for: TET1 Depletion Induces Aberrant CpG Methylation in Colorectal Cancer Cells
Source: PLoS One. 2016 Dec 15;11(12):e0168281. doi: 10.1371/journal.pone.0168281 (PMC5158030; doi:10.1371/journal.pone.0168281)
Supplement: S1 File — Fig A in S1 File. Cell viability assay results from control and TET1 knockdown clones of the indicated CRC cell lines. Fig B in S1 File. Summary of Infinium HumanMethylation450 BeadChip assays with control and TET1 knockdown HCT116 cells. Fig C in S1 File. Heatmap showing the gene expression microarray results from selected 25 genes that were differentially expressed between control and TET1 knockdown clones of Colo320DM cells. Fig D in S1 File. Sequences of the regions analyzed with bisulfite sequencing and shown in Fig 4C and 4F. Fig E in S1 File. Diagram of the HM450 BeadChip results for the entire GRB10 gene region in control and TET1 knockdown clones of Colo320DM cells. Fig F in S1 File. Association between DNA methylation and gene expression in TET1 knockdown Colo320DM cells. Fig G in S1 File. Analysis of DNA methylation and expression of selected genes in control and TET1 knockdown Colo320DM cells. Fig H in S1 File. Attenuated effects of 5-aza-dC (Aza) on gene expression profiles in TET1-depleted Colo320DM cells. (DOC) [file pone.0168281.s001.doc]

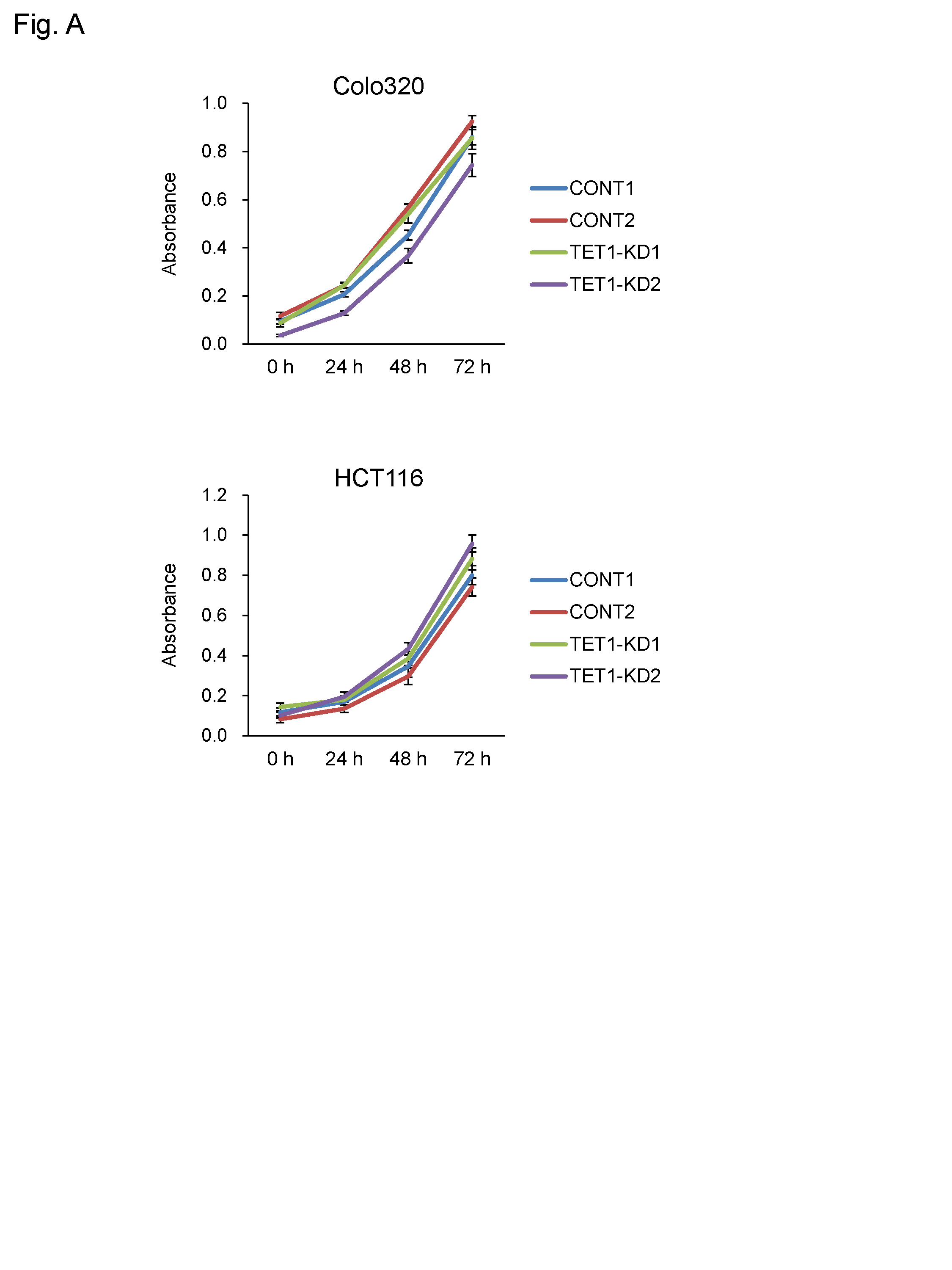


**Fig. A**

Cell viability assay results from control and *TET1* knockdown clones of the indicated CRC cell lines.


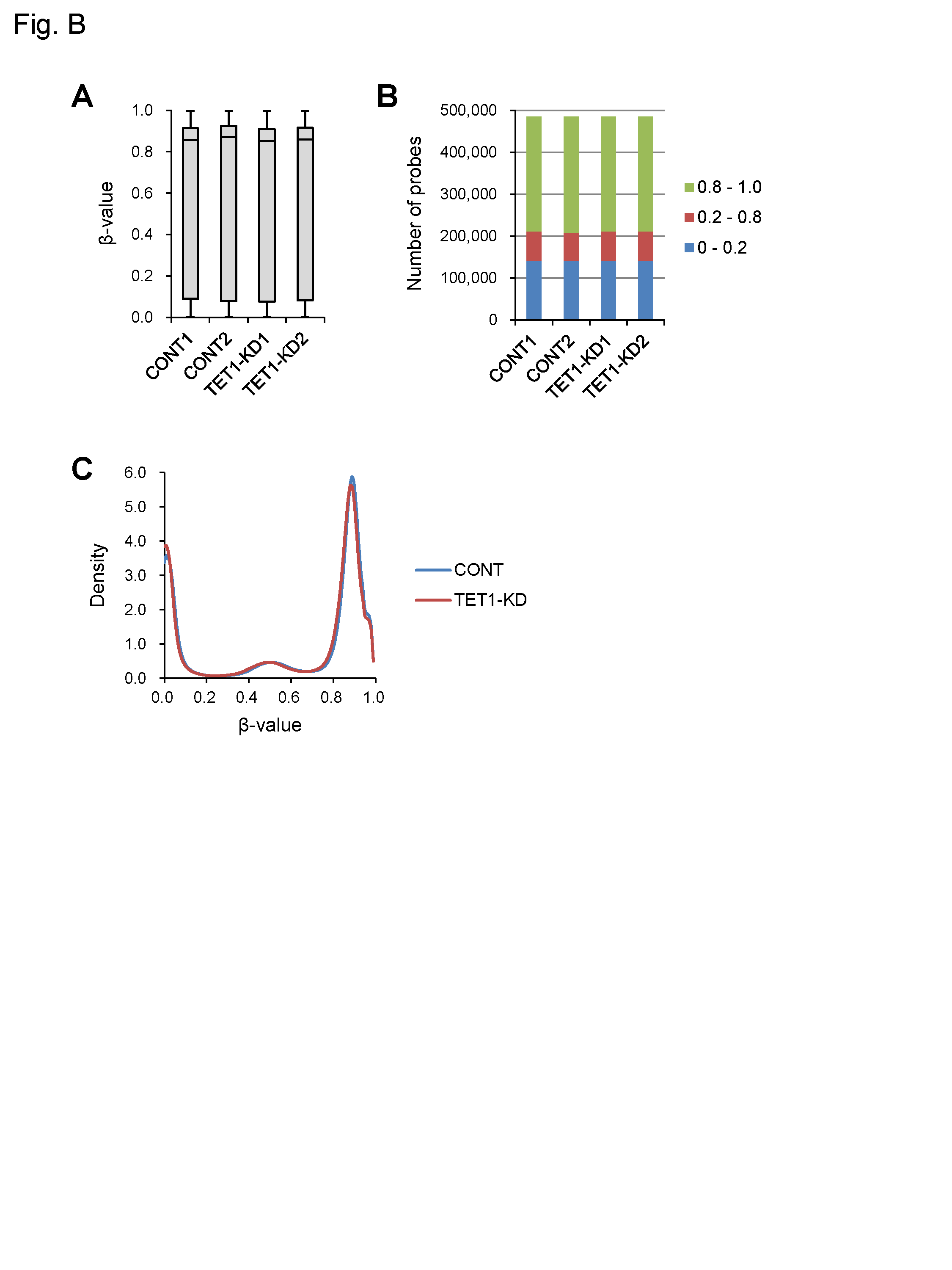


**Fig. B**

Summary of Infinium HumanMethylation450 BeadChip assays with control and *TET1* knockdown HCT116 cells. (A) Box plot showing the β-values of all probe sets in the control and *TET1* knockdown clones. (B) Number of probes with the indicated β-values. (C) Density plots of Infinium BeadChip results from control and *TET1* knockdown clones.


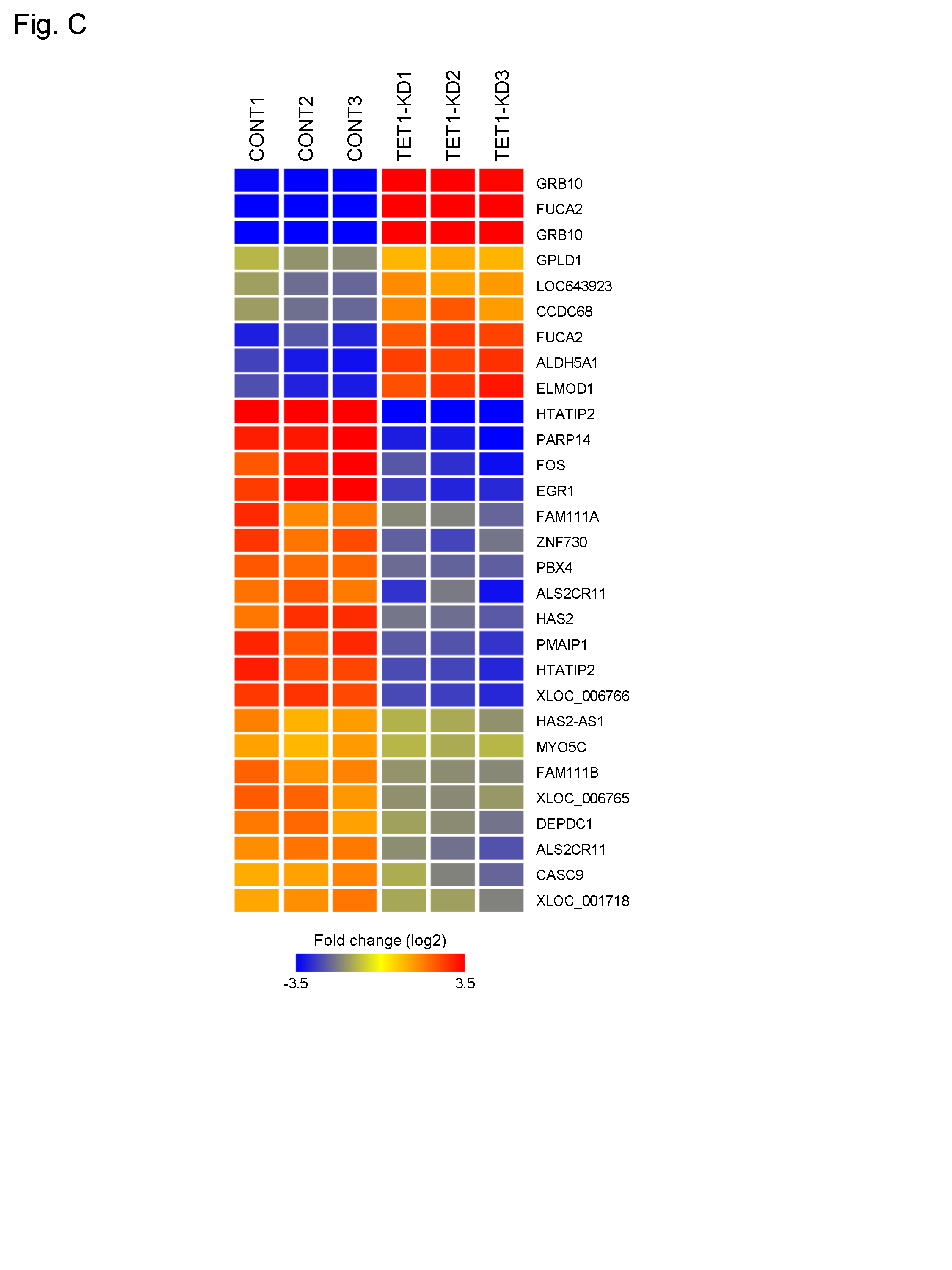


**Fig. C**

Heatmap showing the gene expression microarray results from selected 25 genes that were differentially expressed between control and *TET1* knockdown clones of Colo320DM cells.

**Fig. D**

**HAS2**

GTAGCTCAGAGAAGGCTTTGAATGGCCAATTTCTCTCTCTCCCTCTCCCCCTCCCCGCCTCCCGCTCGCCCGCCCGCCCGCGCTCCCAGTTCCCTCCCCTCAGGGTTCCCCAGTCCACACCTCCCTCTCCACTTCCCTCACCCCCCCACTCCCTCCGCCGCCCTATTAAAACACCCACCAGCTCACTTGTTAAGACCCCCTTAAGTTGGAGGAGGCAGAAGGGCAACAACGGCGGGGAAGGAGAAGTCAAGACGTCTGGAAAGAATTACCCAGTCCTGGCTTCGAGCAGCCCATTGAACCAGAGACTTGAA

**GRB10**

TCGGCCTGCGCAGGCTGCAGAGGCCCCGGCCGCGGCGGACAGGGCCCGGGAGGGAGGCGGGGAGGTCTTGCCGCGCGGCCCCCTGCCCGCCTGCCGCGGGCTCGCTGCAGTCCGAGATCCCGAGCTTCGTTGCCGCCAGCCATAGGACAGTATTATGTAACCATTTTTAATCTTAATTGTTTAATATTATGTAACGAGTAATAACTGTACCCACGGAAACCCGTGACGACGATTCGAGCGAACCTCGCCCGCTCCTTCCTCGCCAGGTGGTGGGCAGCGGGTGTGGGAACCGGTTTGGGAAATGAGTGGATCTGGGCATCCGGGCTACTGCAC

**Fig. D**

Sequences of the regions analyzed with bisulfite sequencing and shown in Fig. 4C and 4F. Underlined sequences represent locations of PCR primers.


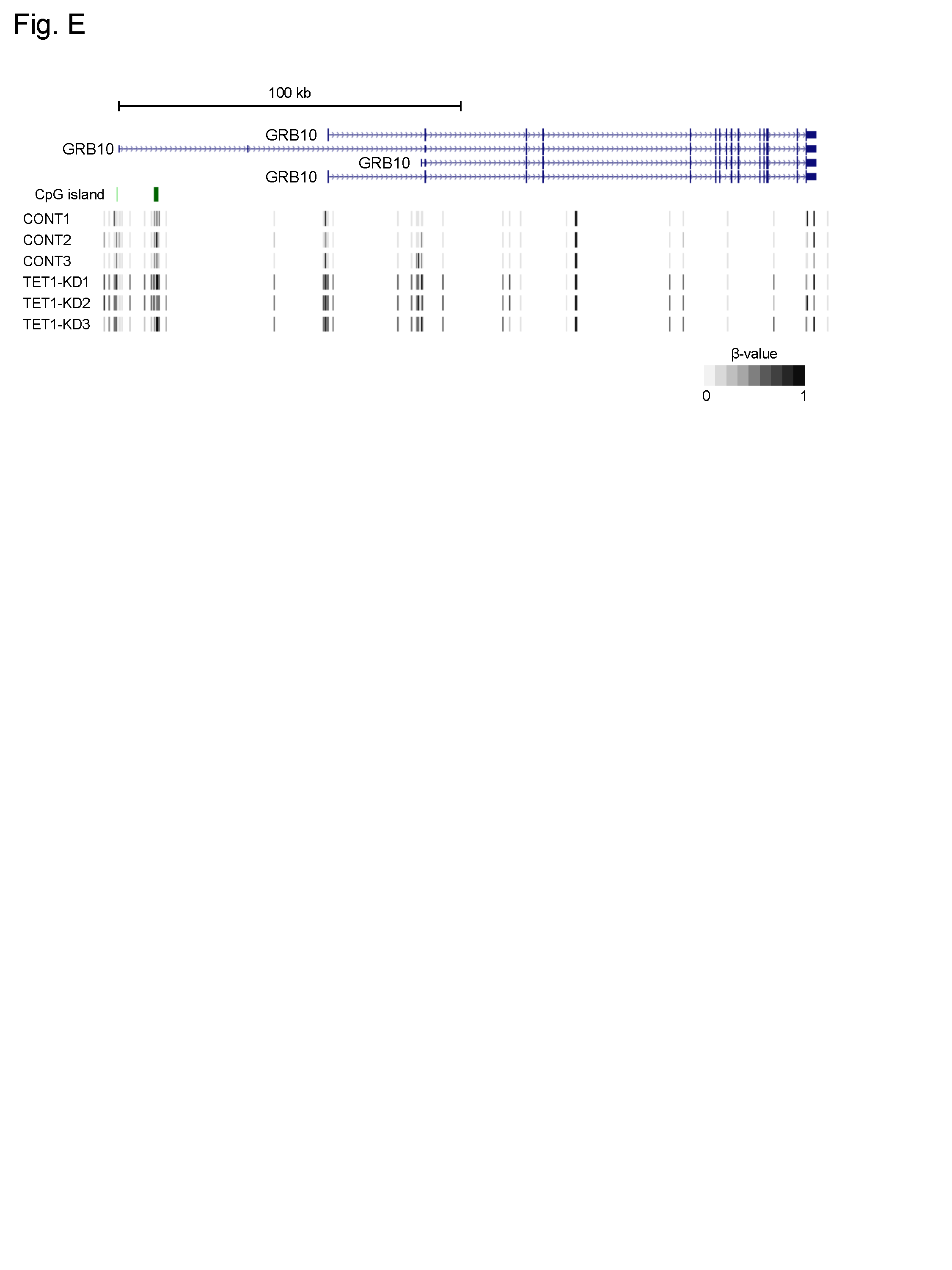


**Fig. E**

Diagram of the BeadChip results for the entire *GRB10* gene region in control and *TET1* knockdown clones of Colo320DM cells.


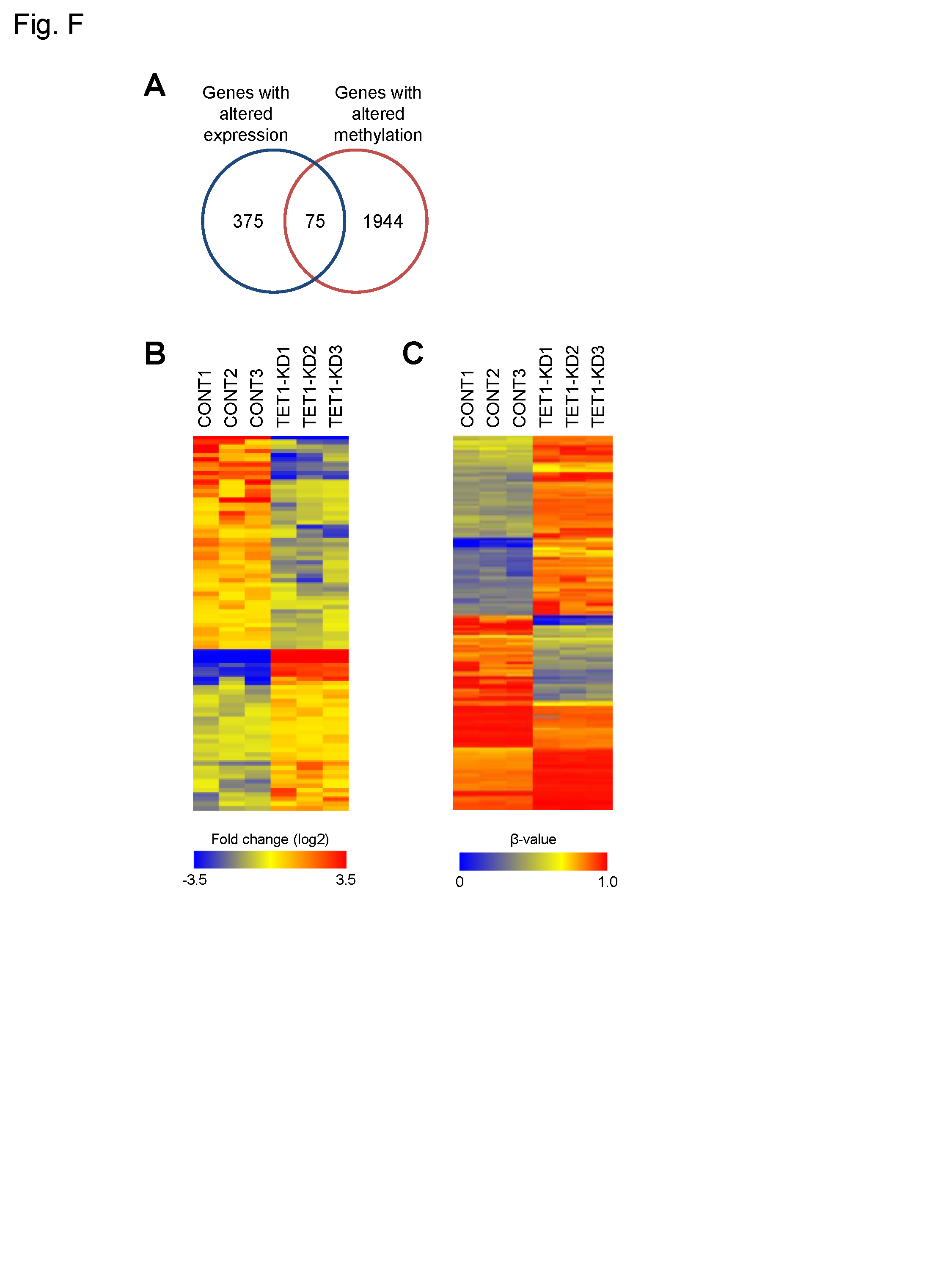


**Fig. F**

Association between DNA methylation and gene expression in *TET1* knockdown Colo320DM cells. (A) Venn diagram for genes with altered DNA methylation (*P* < 0.05) and genes with altered expression (*P* < 0.5) in *TET1* knockdown Colo320DM cells. (B) Heatmap of the expression of the 75 selected genes in (A). (C) Heatmap of the Infinium BeadChip probes for the 75 selected genes.


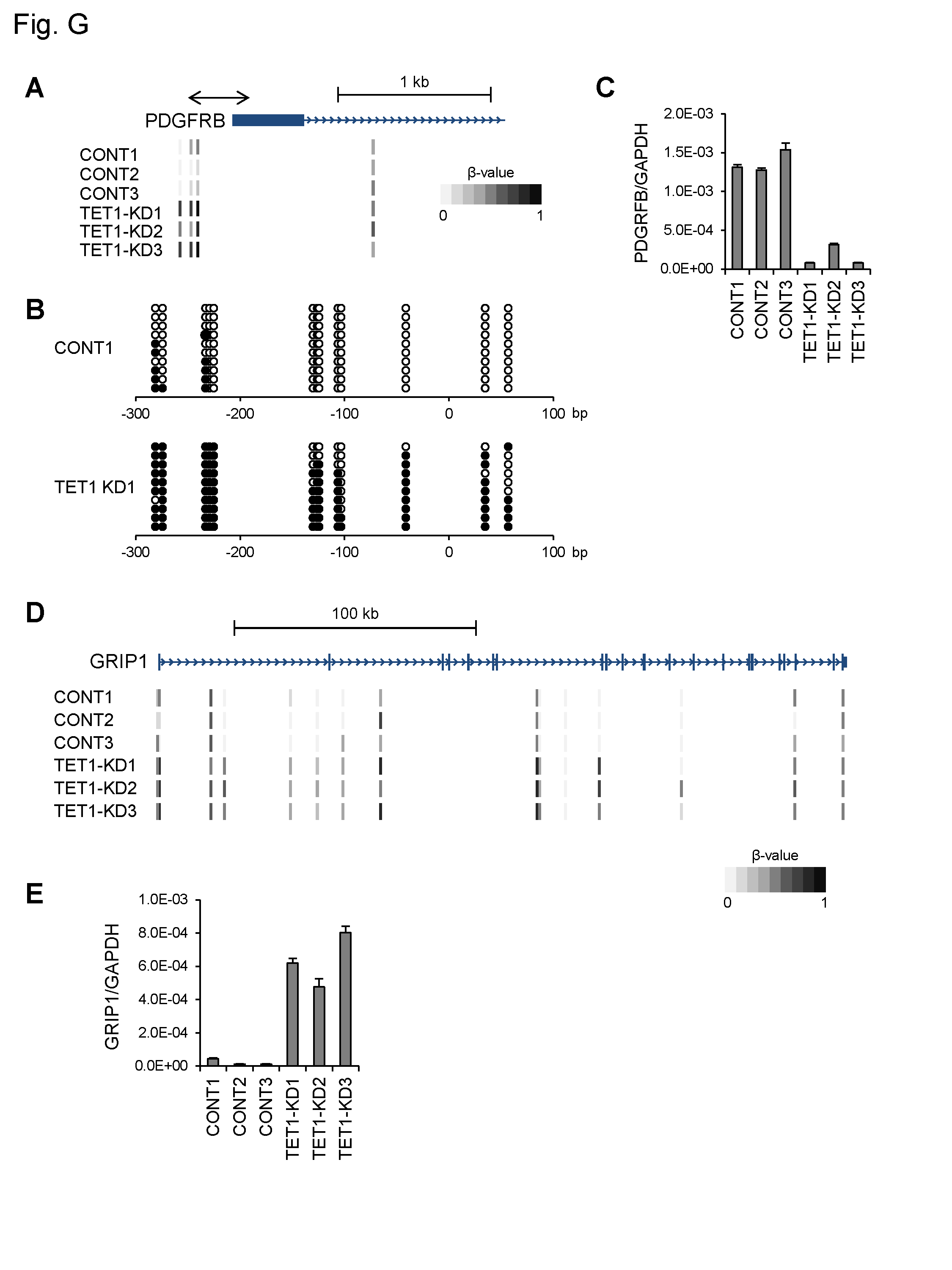


**Fig. G**

Analysis of DNA methylation and expression of selected genes in control and *TET1* knockdown Colo320DM cells. (A) Diagram of Infinium BeadChip results around the transcription start site of *PDFGRB* in control and *TET1* knockdown clones. The region analyzed by bisulfite sequencing is indicated on the top. (B) Bisulfite sequencing analysis results of selected clones. Open and closed circles represent unmethylated and methylated CpG sites, respectively. (C) RT-qPCR results of *PDFGRB* in indicated clones. (D) Infinium BeadChip results for the entire *GRIP1* gene region in control and *TET1* knockdown clones. (E) RT-qPCR results for *GRIP1* in the indicated clones.


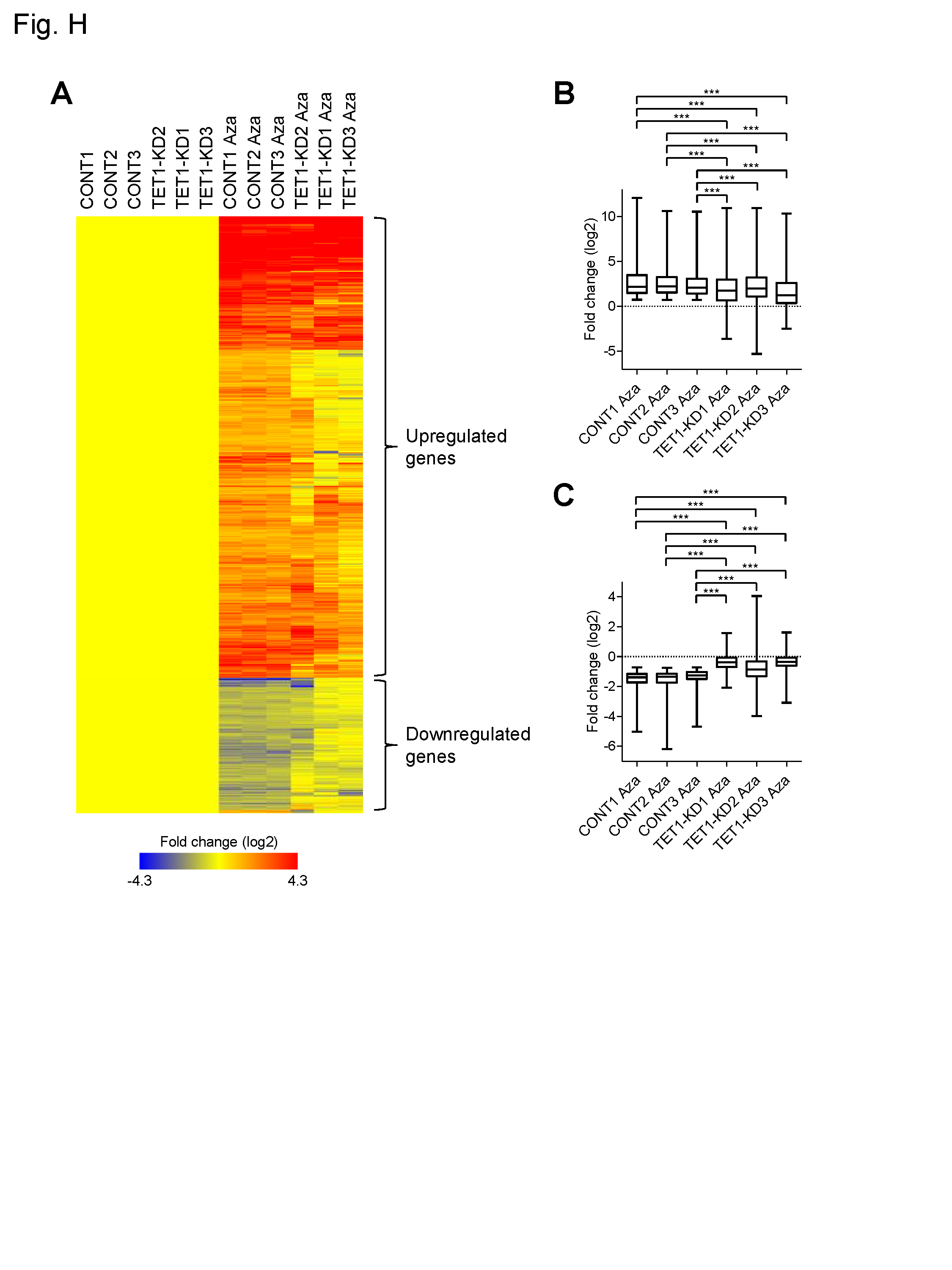


**Fig. H**

Attenuated effects of 5-aza-dC (Aza) on gene expression profiles in *TET1*-depleted Colo320DM cells. (A) Heatmap showing the expression of genes affected by Aza in Colo320DM cells. Genes with altered expression after Aza treatment in control clones were selected, after which hierarchical clustering was performed. (B) Box plot showing the expression levels of genes upregulated by Aza. ****P* < 0.001 (one-way ANOVA with post hoc tests). (C) Box plot showing the expression levels of genes downregulated by Aza. ****P* < 0.001 (one-way ANOVA with post hoc tests).
